# Identification, Screening, and Comprehensive Evaluation of Novel DPP-IV Inhibitory Peptides from the Tilapia Skin Gelatin Hydrolysate Produced Using Ginger Protease

**DOI:** 10.3390/biom12121866

**Published:** 2022-12-13

**Authors:** Wei Liu, Xinyu Wang, Wenning Yang, Xueyan Li, Dongying Qi, Hongjiao Chen, Huining Liu, Shuang Yu, Yanli Pan, Yang Liu, Guopeng Wang

**Affiliations:** 1 Department of Chemistry of Traditional Chinese Medicine, School of Chinese Materia Medica, Beijing University of Chinese Medicine, Beijing 100102, China; 2 Institute of Information on Traditional Chinese Medicine China Academy of Chinese Medical Sciences, Beijing 100700, China; 3 Zhongcai Health (Beijing) Biological Technology Development Co., Ltd., Beijing 101500, China

**Keywords:** ginger protease, gelatin hydrolysate, bioactive peptides, in silico analysis, binding kinetics

## Abstract

Purpose: Inhibition of dipeptidyl peptidase-IV (DPP-IV) is an effective therapy for treating type II diabetes (T2D) that has been widely applied in clinical practice. We aimed to evaluate the DPP-IV inhibitory properties of ginger protease hydrolysate (GPH) and propose a comprehensive approach to screen and evaluate DPP-IV inhibitors. Methods: We evaluated the in vitro inhibitory properties of fish skin gelatin hydrolysates produced by five proteases, namely, neutral protease, alkaline protease, bromelain, papain, and ginger protease, toward DPP-IV. We screened the most potent DPP-IV inhibitory peptide (DIP) using liquid chromatography-tandem mass spectrometry (LC-MS/MS) coupled with in silico analysis. Next, surface plasmon resonance (SPR) technology was innovatively introduced to explore the interactions between DPP-IV and DIP, as well as the IC_50_. Furthermore, we performed oral administration of DIP in rats to study its in vivo absorption. Results: GPH displayed the highest degree of hydrolysis (20.37%) and DPP-IV inhibitory activity (65.18%). A total of 292 peptides from the GPH were identified using LC-MS/MS combined with de novo sequencing. Gly-Pro-Hyp-Gly-Pro-Pro-Gly-Pro-Gly-Pro (GPXGPPGPGP) was identified as the most potent DPP-IV inhibitory peptide after in silico screening (Peptide Ranker and molecular docking). Then, the in vitro study revealed that GPXGPPGPGP had a high inhibitory effect on DPP-IV (IC_50_: 1012.3 ± 23.3 μM) and exhibited fast kinetics with rapid binding and dissociation with DPP-IV. In vivo analysis indicated that GPXGPPGPGP was not absorbed intact but partially, in the form of dipeptides and tripeptides. Conclusion: Overall, the results suggested that GPH would be a natural functional food for treating T2D and provided new ideas for searching and evaluating potential antidiabetic compounds. The obtained GPXGPPGPGP can be structurally optimized for in-depth evaluation in animal and cellular experiments.

## 1. Introduction

The dramatic increase in the incidence of type II diabetes (T2D) is a leading cause of the global disease burden [1]. Dipeptidyl peptidase-IV (DPP-IV) is the key metabolic enzyme in T2D and is widely expressed in most tissues and cells [2]. DPP-IV rapidly inactivates glucagon-like peptide-1 (GLP-1) and glucose-dependent insulinotropic peptide (GIP) in vivo, leading to disorders of insulin secretion and glucose metabolism. Therefore, the inhibition of DPP-IV is considered an effective therapy for treating T2D [3]. A wide variety of DPP-IV inhibitors have been used in treating T2D, such as sitagliptin, vildagliptin, and linagliptin. However, clinical studies have shown that these chemically synthesized DPP-IV inhibitors on the market often cause adverse side effects, including headaches, vertigo, diarrhea, peripheral edema, and abnormal liver function, among others [4,5]. According to the International Diabetes Federation survey, approximately 480 million people are believed to be living with T2D globally [6]. Thus, it is urgent to search for novel, safe, and effective DPP-IV inhibitors. 

In this context, DPP-IV inhibitory peptides obtained from food have garnered much attention owing to their high safety, good stability, and few side effects [7,8]. Evidence is mounting that food-derived proteins and peptides can be used as natural sources of DPP-IV inhibitors, including milk, oats, and rice [9,10,11]. Fish skin, rich in collagen, is a by-product of the processing process and is readily available in large quantities. Previous research has shown that fish skin gelatin hydrolysates, prepared using protease hydrolysis, have DPP-IV inhibitory activity [8,12,13,14]. They contain abundant essential amino acids close to the percentage needed by the human body, which makes them a high-quality raw material for the enzymatic preparation of bioactive peptides [14]. Enzymatic hydrolysis is the most efficient way to prepare peptides from proteins, with the advantages of mild reaction conditions, minimum process complexity, and environmental friendliness [15]. Ginger protease is regarded as a novel collagenase that can hydrolyze collagen macromolecules into small peptides. However, its potential commercial value has not been fully exploited. As compared with other commercial enzymes, ginger protease is reported to have a unique substrate specificity for recognizing Pro at the P_2_ position [16,17]. Thus, we speculated that bioactive peptides might be generated by ginger protease due to its unique hydrolysis site. To our knowledge, no previous study has shown that fish skin gelatin hydrolysates obtained using ginger protease display inhibitory activity toward DPP-IV. 

Although a large number of active peptides and peptidomimetics are known, traditional in vitro methods used to verify the inhibitory activity of inhibitors are usually expressed in terms of IC_50_. On this basis, Swinney and Robert Copeland et al. presented the concept of drug-target binding kinetics and emphasized that it was not the affinity (IC_50_) but the binding kinetics that more clearly reflected the interactions between targets and drugs [18,19,20]. Drug research and development are being gradually dominated by this drug-target binding kinetics strategy, juggling the affinity [21,22]. 

Current studies concerning bioactive peptides are limited to experiments performed in vitro at the cellular level. If the DPP-IV inhibitory peptides validated in vitro are to be transferred to the in vivo situation, the bioavailability of these bioactive peptides must be considered. However, absorption and metabolism in vivo are rarely considered. Although a simulated gastrointestinal fluid is used to simulate the gastrointestinal conditions, it cannot reflect the actual physiological conditions in vivo. Following the theory of serum thermochemistry, only constituents absorbed into the blood have the chance to exert pharmacological bioactivities. It is still unclear whether the polypeptides can reach the targets intact and produce a definite physiological action in the prototype form, partly due to the lack of evidence of absorption and metabolism in vivo in previous studies [23].

Based on the above analysis, we aimed to explore the potential DPP-IV inhibitory activity of fish skin gelatin hydrolysate prepared using ginger protease, aiming to release bioactive DPP-IV inhibitory peptides. We screened the most potent DPP-IV inhibitory peptide from peptide mixtures using liquid chromatography-tandem mass spectrometry (LC-MS/MS) coupled with in silico analysis. Subsequently, we proposed an integrated strategy to evaluate the potential inhibitory activity against DPP-IV, including in vitro assays (affinity and binding kinetics) and in vivo experiments (oral administration in rats).

## 2. Materials and Methods

### 2.1. Materials

Fish skin gelatin from tilapia was supplied by Shanghai Xinxi Biotechnology Co., Ltd. (Shanghai, China). The analysis certificate of gelatin is shown in Supplementary Materials Table S2. Four proteases (neutral protease, alkaline protease, papain, and bromelain) were purchased from Nanning Pangbo Bioengineering Co., Ltd. (Nanjing, China). Recombinant human DPP-IV was provided by ProSpec (Rehovot, Israel). Gly-Pro-pNA was purchased from Dalian Meilun Biological Technology Co., Ltd. (Dalian, China). The synthetic peptides were from Shanghai Top-peptide Biotechnology Co., Ltd. (Shanghai, China).

### 2.2. Preparation of Ginger Protease

The ginger protease was extracted using the previous method [24]. In brief, ginger rhizomes were homogenized in 2 volumes (*w*/*v*) of phosphate buffer. After precipitation with ammonium sulfate, ginger protease was dialyzed, lyophilized, and stored at 20 °C. The enzyme activity is generally expressed in terms of unit (U). Thus, the enzymatic activity was evaluated at 280 nm by determining soluble peptides released from casein [25].

### 2.3. Comparison of Enzymatic Hydrolysis

The enzymatic hydrolysis of fish skin gelatin was conducted with five enzymes (neutral protease, alkaline protease, bromelain, papain, and ginger protease) under optimal conditions (Table 1). Fish skin gelatin (2 g) was dispersed in 40 mL of distilled water. Hydrolysis reactions were initiated by the addition of enzymes (enzyme/substrate [E/S] ratio of 5000 U:1 g) after the pH adjustment. Then, the reaction was terminated in a boiling water bath for 5 min. After centrifugation at 8000 rpm for 15 min, the supernatant was freeze-dried and stored at −20 °C until used.

### 2.4. Degree of Hydrolysis (DH)

The DH was measured by modifying the OPA method proposed [26]. Results were calculated as follows: DH (%) = (h/h_tot_) × 100%, where h_tot_ is the total number of peptide bonds per protein equivalent and h is the number of hydrolyzed bonds.

### 2.5. In Vitro DPP IV Inhibition Assay

The assay was conducted in 96-well microplates, based on the optimized reaction conditions. Sitagliptin was used as the positive control. All the reagents and samples were diluted with 0.1 M Tris-HCl buffer (0.1 M NaCl and 1 mM EDTA, adjusted to pH 8.0). Briefly, 40 μL aliquots of samples were added to 20 μL of 3 mM Gly-Pro-pNA, and the mixture was incubated at 37 ℃ for 10 min. Next, the reaction was initiated by adding 40 μL of 0.4 μg/mL DPP-IV. The absorbance was measured at 405 nm using a Skanlt RE absorbance reader (Thermo Scientific, San Jose, CA, USA) after 30 min of incubation. Results were calculated using the following equation:

$$\mathrm{DPP}-\mathrm{IV}\;\mathrm{inhibition}\;\left(\%\right)=\left[1-\frac{A_{\left(\mathrm{sample}\right)}-A_{\left(\mathrm{sample}\;\mathrm{control}\right)}}{A_{\left(\mathrm{negative}\;\mathrm{reaction}\right)}-A_{\mathrm{negative}\;\mathrm{control}}}\right]\times 100$$

where: A_(sample control)_ contained sample and substrate (Gly-Pro-pNA); A_(negative reaction)_ consisted of DPP-IV and substrate; A_(negative control)_ contained substrate. Lineweaver–Burk plots were used to determine the mode of DPP-IV inhibition of synthetic peptide [27]. IC_50_: the concentration of inhibitors required to inhibit 50% of DPP-IV activity.

### 2.6. Identification of Peptide Sequences by Nano LC-ESI-MS/MS

Since ginger protease hydrolyzed gelatin more efficiently when compared with other proteases, we characterized and identified the peptides in detail. GPH was submitted to nano-liquid chromatography using EASY-nLC 1200 (Thermo Scientific, San Jose, CA, USA) coupled with Q Exactive™ Hybrid Quadrupole-Orbitrap™ Mass Spectrometer (Thermo Scientific, San Jose, CA, USA). The analysis conditions were as follows: pre-column—Acclaim PepMap RPLC C18 300 µm × 5 mm (5 µm, 100 Å; Thermo Scientific, San Jose, CA, USA); analytical column—Acclaim PepMap RPLC C18 150 µm × 150 mm (1.9 µm, 100 Å; Thermo Scientific, San Jose, CA, USA); mobile phase A was 0.1% TFA in 2% ACN (*v*/*v*) and mobile phase B was 0.1% TFA in 80% ACN (*v*/*v*); gradient—0–2 min at 4–8% B, 2–45 min at 8–28% B, 45–55 min at 28–40% B, 55–56 min at 40–95% B and 56–66 min at 95% B; and flow rate—600 nL/min. Data analysis and de novo sequencing were performed using PEAKS studio version 10.6 (Bioinformatics Solutions Inc., Waterloo, ON, Canada).

### 2.7. Screening of Bioactive Peptides Using Bioinformatics Analysis

#### 2.7.1. Peptide Ranker

The Peptide Ranker database (http://distilldeep.ucd.ie/PeptideRanker/, accessed on 5 November 2022) was used for initial screening from the 292 peptides acquired from the GPH (Supplementary Materials Table S1). Following the Peptide Ranker website instructions, the higher score a peptide achieves, the more likely it is to be bioactive. Therefore, the top 25 ranked peptides were selected as potential active peptides. Toxicity was predicted by the ToxinPred server (http://crdd.osdd.net/raghava/toxinpred/, accessed on 5 November 2022).

#### 2.7.2. Molecular Docking

The 2D structures of peptides were drawn in Chem 2D (version 21.0.0), and energy was minimized using the MM2 force field in Chem 3D (version 21.0.0). The LibDock module of Discovery Studio 2019 software (Accelrys Software Inc., San Diego, CA, USA) was used for molecular docking. The crystal structure of DPP-IV was obtained from the protein data bank (PDB ID: 6B1E, with the inhibitor vildagliptin bound in the active site). The radius was set to 12 Å. Docking results were sorted by LibDock score.

### 2.8. Surface Plasmon Resonance Experiments

The DPP-IV was immobilized on a CM5 chip until the response was up to 8000 RU. A 50 μg/mL DPP-IV solution was prepared in acetate buffer (10 mM [pH 5.0]). The running buffer was HEPES buffer (10 mM HEPES [pH 7.4], 150 mM NaCl, 3 mM EDTA, and 0.05% polysorbate 20 [*v*/*v*]). Binding kinetics experiments were carried out by contacting the immobilized DPP-IV with a concentration series of the inhibitor at a flow rate of 30 μL/min using a Biacore T200 instrument (GE Healthcare). The surfaces were regenerated with 0.1 M NaOH after each cycle.

### 2.9. In Vivo Analysis

#### 2.9.1. In Silico Gastrointestinal Digestion

In silico digestion was carried out using Peptide Cutter, available at https://web.expasy.org/peptide_cutter/, accessed on 5 November 2022. The enzymes used were pepsin (pH 1.3) and trypsin.

#### 2.9.2. Oral Administration in Rats

Blood was collected at 0.5 and 1 h, after oral administration, from the abdominal aorta and centrifuged (3000× *g*, 5 min) to acquire plasma. Next, the proteins were precipitated by adding three volumes of 100% ethanol and removed using centrifugation. A volume of 6 μL of supernatant were injected into the LC-MS/MS system, as previously described [16].

### 2.10. Statistical Analysis

All tests were performed in triplicate. Results were shown as mean ± standard deviations (SD). One-way analysis of variance ANOVA was performed using GraphPad Prism version 8.4.0 (GraphPad Software, Inc., La Jolla, CA, USA). Statistical significance was defined as *p* < 0.01.

## 3. Results and Discussion

### 3.1. Comparison of Enzymatic Hydrolysis

Five enzymes were used to hydrolyze fish skin gelatin. After completion of hydrolysis for 4 h, neutral protease, alkaline protease, bromelain, papain, and ginger protease generated hydrolysates that displayed 9.80%, 16.55%, 9.23%, 11.82%, and 20.32% degrees3 of hydrolysis (DH), respectively. The difference in DH values was mainly due to the different hydrolysis sites of enzymes in the enzymatic hydrolysis reaction [28]. Ginger protease hydrolysate (GPH) showed the highest DH among all hydrolysates, suggesting its ability to hydrolyze gelatin more efficiently than other enzymes. 

**Figure 1:**
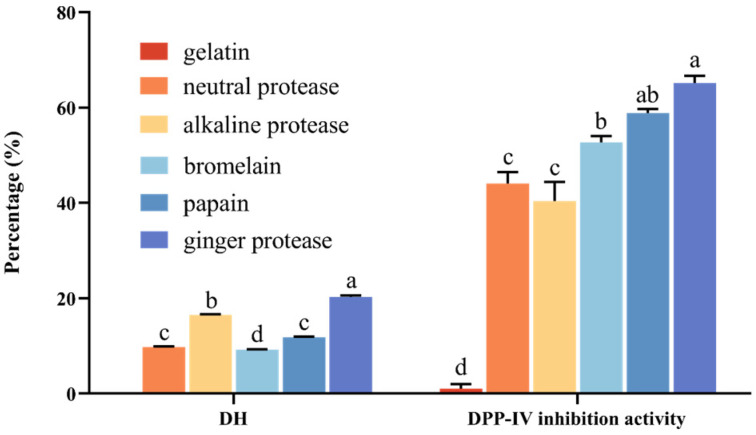
DH and DPP-IV inhibition activity of hydrolysates hydrolyzed using different enzymes. Different letters on bar graphs indicate significant differences (*p* < 0.01).

In addition, gelatin exhibited poor DPP-IV inhibitory activity (<5%), while fish skin gelatin, upon hydrolysis with different enzymes, displayed strong inhibitory activity. This can be attributed to the generation of potential bioactive peptides after hydrolysis. Significant differences in DPP-IV inhibitory activity were observed among hydrolysates (*p* < 0.01; Figure 1). 

Interestingly, GPH with higher DH was more potent in the inhibition of DPP-IV when compared with other hydrolysates. Two probable explanations for the brilliant DPP-IV inhibitory activity of GPH were described as follows. Firstly, GPH displayed the highest DH, and previous research has revealed that ginger protease had overall higher proteolytic activity, which can explain why the hydrolysates produced by ginger protease displayed better effects of hydrolysis when compared with those produced by bromelain and alkaline protease [16,29]. Secondly, the structure and sequence of the peptides produced by ginger protease were consistent with the features of DPP-IV inhibitory peptides. For example, abundant X-Pro-Y type tripeptides were produced by ginger protease for the unique substrate-specific cleavage toward Pro at the P_2_ position. X-Pro-Y type tripeptides with a penultimate Pro from the N-terminus conformed to the structure of DPP-IV inhibitory peptides and have been reported to display DPP-IV inhibitory activity [17,30,31,32,33]. Ginger protease, owing to its unique specificity, has been reported to manufacture a novel wheat gluten hydrolysate that can be used as a functional food for T2D patients. 

Peptidases are usually divided into exopeptidases and endopeptidases. Recent studies have demonstrated that sequential hydrolysis with multiple enzymes was also effective in generating collagen hydrolysates [8,15]. Generally, collagen is first hydrolyzed using endopeptidases and then further hydrolyzed using exopeptidases to release a wider variety of peptides with different amino acid sequences [15]. Since ginger protease is an endopeptidase, we speculated that collagen peptides can be effectively prepared using a two-step enzymatic hydrolysis of ginger protease and exopeptidases.

Ginger protease was the most efficient enzyme for hydrolyzing gelatin, owing to its hydrolytic specificity, but its commercial value has yet to be developed. Therefore, ginger protease was selected for subsequent experiments. Notably, the DPP-IV inhibitory potential of gelatin hydrolysate produced by ginger protease was reported for the first time in the present study.

### 3.2. Identification of Peptide Sequences by Nano LC-ESI-MS/MS Coupled with De Novo Sequencing

The GPH was selected for identifying peptide sequences based on the results presented in Section 3.1. The result showed that a total of 292 peptides were identified with average local confidence (ALC) scores of more than 95%. This range of values indicates the high accuracy of amino acids in the peptide sequence [34,35]. The peptides consisted of approximately 4–15 amino acid residues with molecular weights ranging between 350 and 1500 Da. This integrated method for peptide identification not only dispenses the fractionation and purification of the classical empirical steps, but also prevails over the bioinformatics-driven analysis of in silico hydrolysis [15,23].

The elementary structural unit of the collagen is characterized by a repetitive Gly-X-Y sequence, where X and Y are frequently Pro and Hyp, respectively [36]. The above structure can be observed in identified peptides. In addition, the proportion of each amino acid residue was obtained by dividing its number of presences by the total number. Gly, Pro, Hyp, Leu, and Ala were the most commonly observed amino acids identified within the GPH (Table 2) [37]. Notably, the presence of hydrophobic amino acids (Ala, Gly, Leu, and Pro) can enhance the DPP-IV inhibitory activity of polypeptides [38,39,40].

Previous studies have shown that peptides with a penultimate Pro or Ala residue from the N-terminus can function as DPP-IV inhibitors [41]. The proportion of peptides from the GPH with the above structural features was 38.0%, indicating that GPH may have hypoglycemic effects. Overall, owing to the unique hydrolysis specificity of ginger protease, it contributed to the expansion of the database of active DPP-IV inhibitory peptides.

### 3.3. Screening of Bioactive Peptides Using Bioinformatics Analysis

Traditional methods to screen for DPP-IV inhibitory peptides are time-consuming, labor-intensive, and inefficient. With technological advancements, bioinformatics tools have been widely used to screen for active peptides [42,43].

To begin with, we used the Peptide Ranker website to screen for the potential bioactive peptides (>0.8). Table 3 presents the list of the top 25 ranked peptides. To assess whether the identified peptides can be used as active candidates for drug designing, the prediction of 25 peptides did not show any toxic properties (Table 2). Next, molecular docking was employed to screen and explore the interactions between the 25 candidate peptides and DPP-IV. Overall, this integrated approach, coupling LC-MS/MS with in silico analysis, is efficient for discovering novel bioactive peptides from peptide mixtures using enzymatic hydrolysis [44].

**Figure 2:**
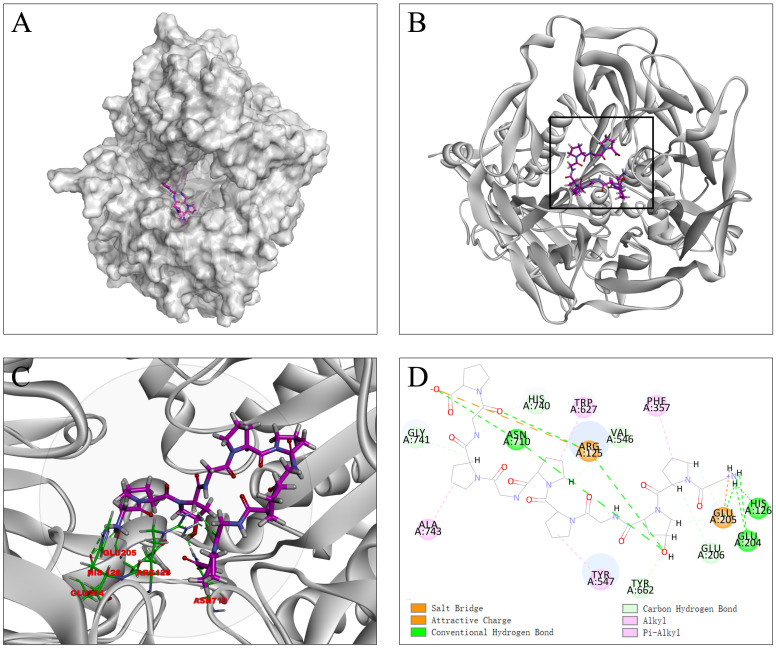
Molecular docking between GPXGPPGPGP and DPP-IV. (**A**) Predicted binding orientation of GPXGPPGPGP (shown in purple) at the ligand-binding site of DPP-IV (shown in grey). (**B**) A 3D view of the best conformation of the GPXGPPGPGP docking with DPP-IV. (**C**) The specific residues of the calculated binding site in DPP-IV. The hydrogen bonds are indicated by green dotted lines. (**D**) A 2D view of the interaction.

Molecular docking-based in silico strategies have been applied recently in the screening and discovery of active food protein-derived peptides [45,46]. The catalytic domain of DPP-IV consists of Ser630, Asp708, and His740. DPP-IV has two substrate-binding pockets (S1 and S2). The S1 pocket includes Tyr547, Ser630, Tyr631, Val656, Trp659, Tyr662, Tyr666, Asn710, Val711, and His740, in which Ser630 and His740 participate in the formation of the catalytic triad. The S2 pocket involves key interactions with Arg125, Glu205, Glu206, Val207, Ser209, Arg358, and Phe 357 [47]. Gly-Pro-Hyp-Gly-Pro-Pro-Gly-Pro-Gly-Pro (GPXGPPGPGP) was bound to the active sites of DPP-IV with the highest docking score of 221.210, suggesting the best inhibitory activity. As shown in Figure 2, the terminal amino group of the GPXGPPGPGP not only made four hydrogen bonds with Arg125 and Glu205 in S1 pockets, but also interacted tightly with Arg204 of DPP-IV. Moreover, both the pyrrole ring of Hyp and the C-terminal Pro residue formed hydrogen bonds with Arg125 and Asn710. As stated above, they facilitated the binding of the substrate to the catalytic site of the enzyme. The C- and N-terminal residues formed salt bridges with Arg125 and Glu205. Hydrophobic interactions were also observed, which have been shown to enhance the inhibition of DPP-IV [48,49].

Recent studies have suggested that peptides containing Pro or Ala residues display potent DPP-IV inhibitory activity. X-Pro is one of the most commonly observed sequences in DPP-IV inhibitory peptides [50]. The Pro residue was mainly bordered by Leu, Val, Phe, Ala, and Gly residues. In this study, X-Pro was observed in the GPXGPPGPGP sequence, and the Pro residue was bordered by Hyp and Gly. Moreover, GPXGPPGPGP primarily consisted of hydrophobic amino acid residues, such as Gly and Pro. As DPP-IV preferentially cleaves the Pro or Ala residue at the penultimate position of the N-terminal, GPXGPPGPGP might behave as a substrate DPP-IV inhibitor.

Post-translational modifications are chemical modifications of post-translational proteins to alter the physicochemical properties of proteins [51]. For example, Hyp, unique in collagen, is a post-translational amino acid resulting from the hydroxylation of Pro residues. Recent studies have indicated that the replacement of Pro with Hyp affected the biological activity of peptides [31,52,53]. Therefore, we compared the binding modes of GPXGPPGPGP and that of GPPGPPGPGP at the active site of DPP-IV. Notably, when compared with GPPGPPGPGP, GPXGPPGPGP with a post-translational hydroxylation displayed a much higher binding affinity for DPP-IV. The interactions between GPXGPPGPGP and DPP-IV indicated that the hydroxyl of Hyp residue showed strong interactions with the Arg125 (S1 pocket) and Asn 710 (S2 pocket) residues of the key amino acids at the active sites of DPP-IV (Figure 2). In contrast, no such interaction was observed between GPPGPPGPGP and DPP-IV. Therefore, further studies are required to comprehensively elucidate the mechanism of DPP-IV inhibition by Pro- hydroxylation. To sum up, GPXGPPGPGP was thus chosen for follow-up evaluations in vitro and in vivo.

### 3.4. Interactions between GPXGPPGPGP and DPP-IV

**Figure 3:**
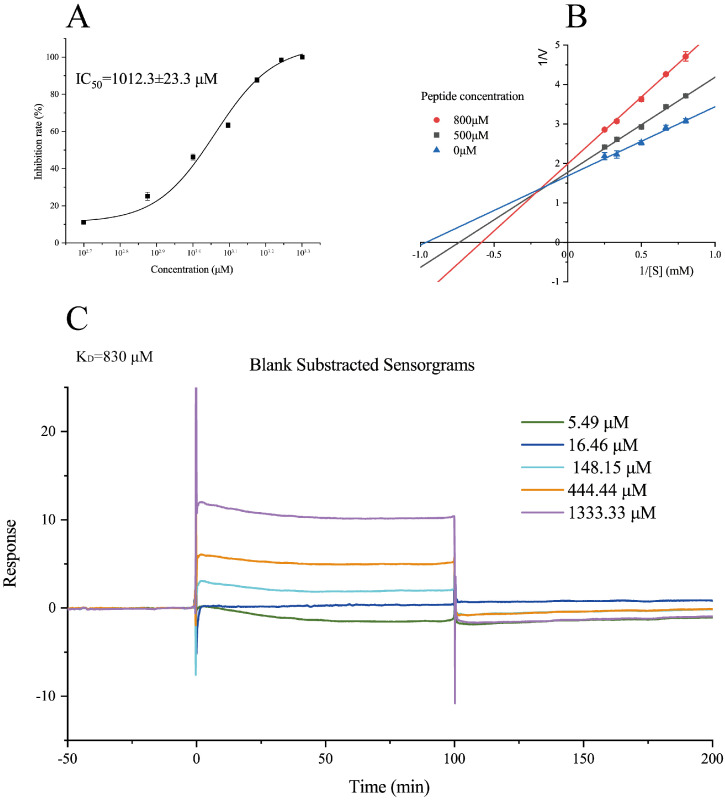
The DPP-IV inhibitory activity of GPXGPPGPG. (**A**) The IC_50_ value. (**B**) Lineweaver-Burk double inverse plots. (**C**): SPR analysis of the interaction.

It was synthesized for subsequent analysis to validate the activity of the GPXGPPGPGP with a penultimate Pro residue from the N-terminus. Sitagliptin is widely used as a clinical drug in treating T2D, and its IC_50_ was found to be 30 ± 0.93 nM in this study. The IC_50_ value was observed for GPXGPPGPGP (1012.3 ± 23.3 μM; Figure 3A).

The Lineweaver-Burk plot was applied to analyze the mode of inhibition on DPP-IV (Figure 3B). All straight lines intersected at the second quadrant, indicating that GPXGPPGPGP showed a mixed-type inhibition mechanism (decreased V_max_ and increased K_m_). The results suggested that GPXGPPGPGP exhibited DPP-IV inhibitory activity by binding to the active site of DPP-IV and beyond the catalytic site.

The traditional strategy for screening active compounds mainly focuses on the optimization of affinity (IC_50_). This method only reflects the equilibrium state of drug-target binding, ignoring the binding and dissociation process of binding kinetics between drugs and targets [19,54].

In this study, we first innovatively introduced binding kinetic parameters to the activity evaluation system of DPP-IV inhibitors. We used the surface plasmon resonance (SPR) technology to explore the binding kinetics between GPXGPPGPGP and DPP-IV. An SPR sensorgram obtained with GPXGPPGPGP over the range 5–1400 μM is depicted in Figure 3C. As the inhibitor concentration increased, the SPR kinetic curve showed a distinct change in trend. GPXGPPGPGP showed high-affinity binding to DPP-IV, characterized by fast binding and dissociation. The equilibrium dissociation constant (K_D_) was measured to be 830 μM, which was close to the IC_50_ measured via in vitro enzyme inhibition assays. The fast binding revealed that the target DPP-IV reached a rapid saturation at low doses. However, the fast dissociation rate suggested a short drug-target residence time, resulting in a short duration of effect in vivo. Therefore, the structure optimization of GPXGPPGPGP will be required to acquire a long drug-target residence time. Altogether, when compared with the classical drug-target binding evaluation method (IC_50_), the whole binding kinetic process allowed a more objective and effective evaluation of the inhibitory activity.

As per the structural characteristics, marketed DPP-IV inhibitors can be divided into two major categories: peptidomimetic and non-peptidomimetic. Vildagliptin and saxagliptin are peptidomimetic drugs discovered on the basis of the substrate-derived Gly-Pro dipeptide scaffold. A previous study reported that the binding of gliptins to DPP-IV displayed strong and extensive interactions with a fast binding and slow dissociation process [55]. They have relatively long residence times on the DPP-IV. Although GPXGPPGPGP did not exhibit very potent DPP-IV inhibitory activity as expected, it has structural features similar to pyrrolidine-based DPP-IV inhibitors. The findings suggested that the selectivity and inhibitory activity of natural peptide GPXGPPGPGP still need improvement, as compared with marketed DPP-IV inhibitors. A series of novel pyrrolidinone analogs can be reasonably designed, synthesized, and validated in subsequent studies [56].

### 3.5. In Vivo Analysis

In silico gastrointestinal digestion was carried out to explore the hydrolysis products of peptides by pepsin (pH 1.3) and trypsin. Pepsin has a preference for cleaving the C-terminal of specific peptide bonds, such as phenylalanine and leucine [57,58]. Trypsin tends to hydrolyze peptide linkages, mainly at the carboxyl side of lysine and arginine residues [58]. We noted that neither of the above two enzymes could hydrolyze GPXGPPGPGP, indicating that GPXGPPGPGP may maintain good structural stability during gastrointestinal digestion. However, it was noteworthy that in silico digestion cannot reflect the actual situation in vivo.

A major advantage of clinical DPP-IV inhibitors is that they can be administered orally. Before reaching the target to exert beneficial effects, bioactive peptides should remain intact after gastrointestinal digestion and enter the blood circulation. However, the in vivo situation after oral administration of bioactive peptides has rarely been considered in published studies [59]. Caco-2 cells have been widely used to study the absorption of peptides across the intestinal epithelia. Some larger peptides, such as Gly-Ala-Hyp-Gly-Leu-Hyp-Gly-Pro, have been shown to pass through the Caco-2 cell monolayer by paracellular diffusion [60]. However, no peptide sequences (longer than or equal to four amino acids) are significantly observed in the blood after the oral administration of collagen hydrolysate or polypeptides.

Therefore, oral administration in rats was performed to explore the absorption of GPXGPPGPGP in vivo. Pro-Hyp, Gly-Pro-Hyp, and Pro-Hyp-Gly were identified in plasma at 0.5 h but not at 1 h, suggesting GPXGPPGPGP can be rapidly absorbed by the body (Table 4). LC-MS/MS results showed GPXGPPGPGP was less able to withstand gastrointestinal digestion and did not remain intact before entering the blood circulation. It was not absorbed intact but partially in the form of dipeptides and tripeptides. Thus, we speculated that was the case for other DPP-IV inhibitory polypeptides. Our study demonstrated that the polypeptides were not suitable for oral administration owing to gastrointestinal digestion.

Many researchers have reported that dipeptides and tripeptides can be successfully absorbed intact through oligopeptide transporter 1 (Pept1) in the gastrointestinal tract [61,62]. Furthermore, the above Hyp-containing oligopeptides can be highly resistant to peptidase degradation in the blood for a short time. Pro-Hyp was not inhibitory against DPP-IV, while Gly-Pro-Hyp was discovered with an inhibitory effect against DPP-IV [32]. Whether Pro-Hyp-Gly has an inhibitory effect on DPP-IV remains to be investigated. We comprehensively analyzed that Gly-Pro-Hyp was mainly responsible for the inhibitory effect on DPP-IV activity in vivo. Thus, gastrointestinal stability, pharmacokinetic, and pharmacodynamic studies in animals should be conducted to provide theoretical guidance for how these food-derived DPP-IV inhibitory peptides exert their glycemic regulatory effects after oral administration.

## 4. Conclusions

Ginger protease has attracted the scientific community because of its unique substrate specificity. This study first demonstrated that GPH is a natural source of bioactive peptides with the potential to inhibit DPP-IV activity in T2D patients. A total of 292 peptides were first identified from GPH, and one novel bioactive peptide GPXGPPGPGP was screened and evaluated by a more efficient and comprehensive strategy, which complements the database of potentially active peptides in T2D. Further structural modification of GPXGPPGPGP is required to improve the inhibitory activity against DPP-IV.

Furthermore, this study establishes a more comprehensive and scientific evaluation index system for screening DPP-4 inhibitors in natural substances, introducing binding kinetic parameters (estimating the binding and dissociation process of binding kinetics between drugs and targets) and absorption characteristics in vivo (revealing the form of bioactive peptides absorbed after oral administration) to the activity evaluation system of DPP-IV inhibitors. On the whole, this study provides new research ideas and a material basis for the development of new drugs for T2D.

## Figures and Tables

**Table 1:** Hydrolysis conditions.

Enzymes	pH	Temperature/°C	Time (h)
neutral protease	7.0	55	4
alkaline protease	9.0	55	4
papain	6.0	55	4
bromelain	6.0	55	4
ginger protease	6.0	55	4

**Table 2:** Amino acid composition of GPH.

Amino Acid	Number of Residues	Percentage (%)
Gly	679	30.0
Pro	537	23.7
Hyp	298	13.2
Leu	164	7.2
Ala	160	7.1
Phe	90	4.0
Val	72	3.2
Arg	69	3.0
Lys	45	2.0
Gln	34	1.5
Asp	31	1.4
Ser	26	1.1
Met	15	0.7
Asn	13	0.6
Thr	11	0.5
Tyr	9	0.4
Glu	5	0.2
Trp	4	0.2
His	4	0.2

**Table 3:** Screening peptides by peptide ranker and molecular docking.

Num	Peptide Sequence	Formula	Length	Molecular Weight	Peptide Ranker	-Libdock Score	ToxinPred	ALC	t_R_
1	GPXGPPGPGP	C_38_H_56_N_10_O_12_	10	844.403	0.948	221.210	Non-toxicity	96	16.19
2	WFXGPR	C_38_H_50_N_10_O_8_	6	774.376	0.987	212.183	Non-toxicity	96	13.55
3	GPPGPXGPGP	C_38_H_56_N_10_O_12_	10	844.403	0.948	209.382	Non-toxicity	97	15.15
4	GPXGPXGPGX	C_38_H_56_N_10_O_14_	10	876.383	0.948	205.059	Non-toxicity	97	11.35
5	GPXGPXGPGP	C_38_H_56_N_10_O_13_	10	860.393	0.948	204.386	Non-toxicity	99	13.76
6	GFXGFQ	C_32_H_41_N_7_O_9_	6	667.292	0.951	202.225	Non-toxicity	97	36.35
7	GPXGPXGGPR	C_39_H_61_N_13_O_13_	10	919.441	0.950	198.751	Non-toxicity	99	8.53
8	GPXGPGXGMP	C_38_H_58_N_10_O_13_S	10	894.381	0.954	194.667	Non-toxicity	97	18.2
9	GPPGPPGPGP	C_38_H_56_N_10_O_11_	10	828.413	0.948	192.787	Non-toxicity	97	18.68
10	FXGFQ	C_30_H_38_N_6_O_8_	5	610.270	0.971	189.562	Non-toxicity	95	33.49
11	FFXGPK	C_36_H_49_N_7_O_8_	6	707.359	0.957	189.253	Non-toxicity	99	17.38
12	FXGMY	C_30_H_39_N_5_O_8_S	5	629.247	0.975	187.274	Non-toxicity	96	34.32
13	LXGPLF	C_33_H_50_N_6_O_8_	6	658.364	0.963	185.441	Non-toxicity	99	44.96
14	FXGPR	C_27_H_40_N_8_O_7_	5	588.297	0.967	178.364	Non-toxicity	97	11.16
15	FXGFM	C_30_H_39_N_5_O_7_S	5	613.252	0.994	177.207	Non-toxicity	95	46.23
16	GFXGPR	C_29_H_43_N_9_O_8_	6	645.319	0.961	173.383	Non-toxicity	97	11.97
17	FXGPF	C_30_H_37_N_5_O_7_	5	579.264	0.994	165.885	Non-toxicity	95	41.44
18	SGPLF	C_25_H_37_N_5_O_7_	5	519.269	0.953	165.737	Non-toxicity	96	37.19
19	LXGFM	C_27_H_41_N_5_O_7_S	5	579.268	0.974	163.263	Non-toxicity	98	40.22
20	FXGGLM	C_29_H_44_N_6_O_8_S	6	636.289	0.963	162.622	Non-toxicity	96	38.19
21	LGPF	C_22_H_32_N_4_O_5_	4	432.237	0.970	148.961	Non-toxicity	99	37.6
22	LPGPF	C_27_H_39_N_5_O_6_	5	529.290	0.973	148.511	Non-toxicity	99	41.31
23	LXGPF	C_27_H_39_N_5_O_7_	5	545.280	0.973	148.113	Non-toxicity	99	34.29
24	FXGAP	C_24_H_33_N_5_O_7_	5	503.233	0.948	146.510	Non-toxicity	97	16.05
25	GPLF	C_22_H_32_N_4_O_5_	4	432.237	0.973	136.346	Non-toxicity	95	34.93

X: Hyp.

**Table 4:** Identification of oligopeptides (dipeptides and tripeptides) in rat plasma after oral administration of GPXGPPGPGP.

Num.	Peptide	0.5 h	1 h
1	PX	+	−
2	GPX	+	−
3	PXG	+	−
4	GPXGPPGPGP	−	−

X: Hyp. +: detected; −: not detected.

## Data Availability

All data included in this study are available upon request by contact with the corresponding author.

## Supplementary Materials

The following supporting information can be downloaded at: https://www.mdpi.com/article/10.3390/biom12121866/s1, Table S1: Peptides from the GPH identified by nano LC-MS/MS; Table S2: The analysis certificate of gelatin supplied by Shanghai Xinxi Biotechnology Co.
