# Supplementary material for: Identification, Screening, and Comprehensive Evaluation of Novel DPP-IV Inhibitory Peptides from the Tilapia Skin Gelatin Hydrolysate Produced Using Ginger Protease
Source: Biomolecules. 2022 Dec 13;12(12):1866. doi: 10.3390/biom12121866 (PMC9775409; doi:10.3390/biom12121866)
Supplement: Supplementary file 1 [file biomolecules-12-01866-s001.zip › biomolecules-2049290-supplementary.pdf]

Table S1 Peptides from the GPH identified by nano LC-MS/MS

No.	Peptide sequence	ALC	Mass/Da	length	t_R	PTM
1	FXGFM	96	613.2521	5	46.23	Pro->Hyp
2	FXGPF	96	579.2643	5	41.44	Pro->Hyp
3	WFXGPR	97	774.3763	6	13.55	Pro->Hyp
4	FXGMY	97	629.247	5	34.32	Pro->Hyp
5	LXGFM	99	579.2678	5	40.22	Pro->Hyp
6	GPLF	97	432.2372	4	34.93	
7	LXGPF	99	545.28	5	34.29	Pro->Hyp
8	LPGPF	97	529.29	5	41.31	
9	FXGFQ	97	610.2701	5	33.49	Pro->Hyp
10	LGPF	95	432.2372	4	37.6	
11	FXGPR	99	588.2971	5	11.16	Pro->Hyp
12	FXGGLM	96	636.2892	6	38.19	Pro->Hyp
13	LXGPLF	99	658.3641	6	44.96	Pro->Hyp
14	GFXGPR	97	645.3185	6	11.97	Pro->Hyp
15	FFXGPK	95	707.3593	6	17.38	Pro->Hyp
16	GPXGPGXGMP	97	894.3807	10	18.2	Pro->Hyp
17	SGPLF	95	519.2693	5	37.19	
18	GFXGFQ	96	667.2916	6	36.35	Pro->Hyp
19	GPXGPXGGPR	98	919.4413	10	8.53	Pro->Hyp
20	FXGAP	96	503.233	5	16.05	Pro->Hyp
21	GPXGPXGPGP	99	860.3929	10	13.76	Pro->Hyp
22	GPXGPPGPGP	99	844.403	10	16.19	Pro->Hyp
23	GPPGPPGPGP	99	828.413	10	18.68	
24	GPXGPXGPGX	97	876.3829	10	11.35	Pro->Hyp
25	GPPGPXGPGP	95	844.403	10	15.15	Pro->Hyp
26	AFXGPR	95	659.3342	6	11.68	Pro->Hyp
27	GPXGPXGGRP	95	919.4413	10	8.56	Pro->Hyp
28	APDPF	97	545.2485	5	29.79	
29	AGFXGPR	98	716.3556	7	13.01	Pro->Hyp
30	FXGLR	97	604.3284	5	21.32	Pro->Hyp
31	AGGFXGPR	97	773.3771	8	13.46	Pro->Hyp
32	GPXGPHFXKGP	95	1118.541	11	9.83	Pro->Hyp
33	GPXGGPXGPXGPGP	96	1184.5315	14	16.37	Pro->Hyp
34	SGFXGPR	97	732.3505	7	12.91	Pro->Hyp
35	W(+42.01)VPGPM	96	727.3363	6	35.62	Acetylation (N-term)
36	LXGFXGPR	96	871.4453	8	25.74	Pro->Hyp
37	GPXGPGXGPGXGAP	98	1158.5156	14	14.73	Pro->Hyp
38	LPFR	95	531.3169	4	19.19	
39	PGPGMP	98	554.2523	6	16.39	
40	FXGPA	98	503.233	5	17.11	Pro->Hyp
41	FGXGPR	99	645.3185	6	11.48	Pro->Hyp
42	GPXGGPXGPPG	98	917.4144	11	12.94	Pro->Hyp
43	GPXGGPXGPXG	96	933.4044	11	10.97	Pro->Hyp
44	AGPXGPXGPGP	96	931.4301	11	14.47	Pro->Hyp
45	GPLFR	97	588.3383	5	18.09	

46	GPXGPPG	96	593.2759	7	9.72	Pro->Hyp
47	APDPFR	98	701.3496	6	20.1	
48	GPPVPGPLGP	98	886.4912	10	35	
49	FXGGLR	96	661.3498	6	17.12	Pro->Hyp
50	GPXGPXGLGXGAP	96	1117.5256	13	22.57	Pro->Hyp
51	AXGFXGPK	98	801.3922	8	13.6	Pro->Hyp
52	GPXGPXGPGXGAP	99	1101.4944	13	15.38	Pro->Hyp
53	APDPGPGMP	97	837.3691	9	20.93	
54	GPXGPXGGPSPXG	95	1117.4893	13	13.31	Pro->Hyp
55	GAPDPGPGMP	97	894.3905	10	22.9	
56	AAFXGPR	98	730.3713	7	12.9	Pro->Hyp
57	GPXGPXGGKP	95	891.4351	10	8.03	Pro->Hyp
58	GPXGPXGGRVGPXGAP	96	1413.6853	16	16.1	Pro->Hyp
59	GPXGPGXGAGP	96	891.3987	11	11.52	Pro->Hyp
60	GPXGLXGPXGPA	96	1060.5042	12	24.62	Pro->Hyp
61	VPGPLF	98	628.3584	6	44.37	
62	XPGPPGP	97	633.3073	7	25.2	Pro->Hyp
63	GPXGPXGRXGAP	96	1103.5212	12	10.17	Pro->Hyp
64	GPXGPXGPA	98	777.3558	9	11.45	Pro->Hyp
65	GPXGPPGPA	97	761.3658	9	13.91	Pro->Hyp
66	LFXGPR	98	701.3811	6	16.19	Pro->Hyp
67	GPXGGPXGPXGAP	98	1101.4944	13	14.85	Pro->Hyp
68	GPXGLXGPGA	95	850.4086	10	18.36	Pro->Hyp
69	GPXGGGPXPGXGAP	95	1158.5156	14	13.16	Pro->Hyp
70	FXGPK	98	560.2909	5	10.29	Pro->Hyp
71	FXGAR	97	562.2814	5	9.11	Pro->Hyp
72	GPXGFGXGAP	98	884.3929	10	23	Pro->Hyp
73	GFXGPK	98	617.3124	6	11.08	Pro->Hyp
74	NPXGPXGPPG	96	917.4144	10	14.07	Pro->Hyp
75	PGPL	97	382.2216	4	34.87	
76	A(+42.01)XGPPGP	97	649.3022	7	21	Acetylation (N-term); Pro->Hyp
77	AAPDPGPGMP	97	908.4062	10	22.73	
78	AXGPVF	99	602.3015	6	29.55	Pro->Hyp
79	W(+42.01)VPGP	97	596.2958	5	25.9	Acetylation (N-term)
80	FXGGPXGPT	95	857.3821	9	21.77	Pro->Hyp
81	VGFM	95	452.2094	4	33.84	
82	N(+.98)PGPGMP	95	669.2792	7	19.49	Deamidation (NQ)
83	AGFXGPK	97	688.3495	7	12.03	Pro->Hyp
84	GPXGPGP	98	593.2759	7	9.74	Pro->Hyp
85	GPXGPYGPXGAP	97	1094.4934	12	29.01	Pro->Hyp
86	GPXGLXGPT	96	823.3977	9	20.04	Pro->Hyp
87	LXGGPXGPXGAP	96	1060.5042	12	20.21	Pro->Hyp
88	GPXGPXGGAXGAP	98	1075.4785	13	12.59	Pro->Hyp
89	LXGPLL	97	624.3797	6	38.51	Pro->Hyp
90	GPVF	98	418.2216	4	27.58	
91	PGPGLP	95	536.2958	6	35.54	

92	LXGPL	99	511.2957	5	23.75	Pro->Hyp
93	LPGPL	99	495.3057	5	29.17	
94	GPXGPXGXGPGPS	95	1117.4893	13	13.83	Pro->Hyp
95	FXGGAP	97	560.2545	6	16.21	Pro->Hyp
96	GPXGPXGAP	98	777.3558	9	10.94	Pro->Hyp
97	GPXGPPGAP	98	761.3658	9	14.35	Pro->Hyp
98	GPXGGLXGPT	96	880.4192	10	17.61	Pro->Hyp
99	FXGLA	98	519.2643	5	31.72	Pro->Hyp
100	GPXGPGXGAP	99	834.3773	10	11.61	Pro->Hyp
101	GPPGPGXGAP	98	818.3873	10	13.5	Pro->Hyp
102	LN(+.98)FXGPR	95	816.408	7	25.18	Deamidation (NQ); Pro->Hyp
103	FGXGAP	95	560.2545	6	18.31	Pro->Hyp
104	GPXGFGXPQ	96	884.3929	9	22.47	Pro->Hyp
105	AGGPXGPXGAP	99	905.4144	11	12.78	Pro->Hyp
106	GPXGPXGGVP	96	862.4086	10	16.82	Pro->Hyp
107	GHRGF	95	572.2819	5	7.43	
108	AGPXGPXGAP	99	848.3929	10	13.11	Pro->Hyp
109	GPPVPGPLK	95	860.512	9	21.24	
110	XGPR	99	441.2286	4	10.91	Pro->Hyp
111	QGGGPXGPXGAP	97	1019.4573	12	12.33	Pro->Hyp
112	A(+42.01)XGPLR	97	667.3604	6	13.85	Acetylation (N-term); Pro->Hyp
113	GPXGPXGAGXPQ	95	1075.4785	12	12.05	Pro->Hyp
114	AGGPLGPR	95	723.4027	8	10.76	
115	GPLFK	95	560.3322	5	17.07	
116	LXGPPGP	99	649.3386	7	21.06	Pro->Hyp
117	LXGPXGP	98	665.3286	7	17.46	Pro->Hyp
118	LPGPPGP	98	633.3486	7	25.26	
119	QGGPXGPXGAP	98	962.4359	11	12.59	Pro->Hyp
120	PGLP	98	382.2216	4	34.34	
121	LXGFXGPK	95	843.4391	8	25.04	Pro->Hyp
122	GPXGAP	97	510.2389	6	22.75	Pro->Hyp
123	D(+42.01)PGPGP	96	580.2493	6	15.98	Acetylation (N-term)
124	LXGFT	97	549.2749	5	28.43	Pro->Hyp
125	LXGFQ	99	576.2858	5	26.66	Pro->Hyp
126	GPPVPGP	98	619.3329	7	20.35	
127	N(+42.01)PGPGP	97	579.2653	6	15.98	Acetylation (N-term)
128	GPXGGPXAGP	96	834.3773	10	12.69	Pro->Hyp
129	FXGGPT	96	590.2651	6	16.22	Pro->Hyp
130	K(+42.01)XGPPGP	96	706.36	7	23.92	Acetylation (N-term); Pro->Hyp
131	FXGVR	97	590.3127	5	14.64	Pro->Hyp
132	GPXGLGXGAP	97	850.4086	10	18.89	Pro->Hyp
133	AGPLGPR	96	666.3813	7	10.66	
134	AAGPXGPGP	98	735.3502	9	11.53	Pro->Hyp
135	GFXGLQ	97	633.3073	6	10.66	Pro->Hyp

136	GPXGAGXGPGXPQ	96	1132.5	13	12.07	Pro->Hyp
137	XGGPR	99	498.2501	5	12.58	Pro->Hyp
138	EFXGPR	95	717.3397	6	11.79	Pro->Hyp
139	LPGLP	98	495.3057	5	30.24	
140	GAGAGPXGPXGAP	96	1033.4729	13	13.54	Pro->Hyp
141	QGGGPXGPXAGP	96	1019.4573	12	11.82	Pro->Hyp
142	FGXLQ	96	576.2858	5	28.26	Pro->Hyp
143	TGFXGPR	97	746.3662	7	13.5	Pro->Hyp
144	A(+42.01)XGPPGAP	95	720.3393	8	21.53	Acetylation (N-term); Pro->Hyp
145	K(+42.01)PGPL	96	552.3271	5	33.65	Acetylation (N-term)
146	PVPGMP	96	596.2992	6	37.63	
147	LXGPLR	98	667.3968	6	14.02	Pro->Hyp
148	GPLGPR	98	595.3442	6	9.69	
149	GPXGVGPXGAP	98	933.4457	11	16.55	Pro->Hyp
150	GPXGGLXGPQ	95	907.4301	10	16.46	Pro->Hyp
151	GPLGGPR	96	652.3656	7	9.83	
152	MGPPAP	95	568.2679	6	30.61	
153	GPXGPYGPQ	98	884.3979	9	21.97	Pro->Hyp
154	LFXGPK	98	673.3749	6	14.75	Pro->Hyp
155	QXGGADGRXGPXGPG	95	1363.5969	15	9.43	Pro->Hyp
156	A(+42.01)GPL	96	398.2165	4	27.79	Acetylation (N-term)
157	RGFXGAR	96	775.404	7	8.6	Pro->Hyp
158	LXGGLP	95	568.3171	6	26.05	Pro->Hyp
159	GPXGPXGEKGSGP	97	1164.5312	13	8.82	Pro->Hyp
160	DGPGPPGAP	95	763.35	9	22	
161	LXGPGLP	97	665.3699	7	26.7	Pro->Hyp
162	ERFXGPR	97	873.4407	7	10.49	Pro->Hyp
163	APDPLR	98	667.3653	6	13.27	
164	GPXGPXGHSGPQ	95	1115.4897	12	8.22	Pro->Hyp
165	LGPL	97	398.2529	4	27.02	
166	GPXGGLVGPK	95	893.4921	10	14.26	Pro->Hyp
167	FALR	97	505.3012	4	18.04	
168	FGLXGAP	95	673.3386	7	34.49	Pro->Hyp
169	LXGGPXGAP	95	793.3871	9	16.72	Pro->Hyp
170	XGGLR	96	514.2814	5	13.05	Pro->Hyp
171	NAGPXGPXGAP	98	962.4359	11	13.1	Pro->Hyp
172	GAXGQDGRXGPXGPG	95	1363.5969	15	9.43	Pro->Hyp
173	H(+42.01)PDPLR	95	775.3976	6	16.51	Acetylation (N-term)
174	XGLR	95	457.2599	4	15.44	Pro->Hyp
175	ALXGPPGAP	97	791.4128	9	22.95	Pro->Hyp
176	ALXGPXGAP	96	807.4028	9	18.68	Pro->Hyp
177	K(+42.01)XGPXGPA	95	793.3871	8	19.87	Acetylation (N-term); Pro->Hyp
178	GLPGPPGAP	97	761.4072	9	28.06	
179	GLXGPXGAP	95	793.3871	9	18.82	Pro->Hyp
180	SXGGSFGPA	97	791.34	9	20.09	Pro->Hyp

181	GPALGXGPA	95	751.3815	9	17.24	Pro->Hyp
182	AFGPXGGAP	96	785.3658	9	19.94	Pro->Hyp
183	GPXGGRVGPXGAP	98	1146.5684	13	12.39	Pro->Hyp
184	LXGLR	99	570.344	5	14.85	Pro->Hyp
185	LXGGLR	97	627.3655	6	13.37	Pro->Hyp
186	VAXGPPGP	95	706.36	8	23.38	Pro->Hyp
187	AXGLXGPQ	95	767.3715	8	15.37	Pro->Hyp
188	AXGPVLR	98	724.4182	7	12.11	Pro->Hyp
189	LXGAP	97	469.2487	5	12.05	Pro->Hyp
190	AXGGADGRXGPXGGP	95	1306.5754	15	8.95	Pro->Hyp
191	A(+42.01)GPR	97	441.2336	4	12.17	Acetylation (N-term)
192	APGVPGP	96	593.3173	7	16.08	
193	VXGPGP	98	538.2701	6	11.31	Pro->Hyp
194	K(+42.01)GPVF	96	588.3271	5	37.61	Acetylation (N-term)
195	AXGVGPXGAP	98	850.4086	10	14.36	Pro->Hyp
196	NFXGGAP	95	674.2974	7	19.38	Pro->Hyp
197	LXGPPGAP	99	720.3757	8	20.73	Pro->Hyp
198	LPGPPGAP	99	704.3857	8	24.92	
199	LXGPXGAP	98	736.3657	8	17.14	Pro->Hyp
200	LXGPLK	99	639.3906	6	13.43	Pro->Hyp
201	LPGPLK	98	623.4006	6	16.33	
202	LAGPL	96	469.29	5	23.37	
203	FTGLR	97	592.3333	5	15.39	
204	LXGAL	98	485.28	5	21.27	Pro->Hyp
205	AGK(+43.01)XGPXGAP	96	922.4451	10	20.54	Carbamoylation; Pro->Hyp
206	QXGGQDGRPGPXGGP	98	1347.6069	14	11.65	Pro->Hyp
207	PVPGP	95	465.2587	5	28.64	
208	RGFXGGAR	96	832.4254	8	8.1	Pro->Hyp
209	FSGLD	98	537.2435	5	23.74	
210	LXGAGP	97	526.2701	6	10.69	Pro->Hyp
211	GPLGXGAP	97	680.3444	8	19.63	Pro->Hyp
212	SPGPDGKAGPA	95	952.4614	11	8.74	
213	AXGQDGRXGPXGGP	99	1306.5754	14	9.46	Pro->Hyp
214	LXGLXGAP	95	752.397	8	31.1	Pro->Hyp
215	GPXGRVGPXGAP	98	1089.5469	12	12.46	Pro->Hyp
216	GLPGXGPPA	96	777.3972	9	23.46	Pro->Hyp
217	VXGPGXGAP	97	779.3715	9	13.11	Pro->Hyp
218	LLGPXG	95	568.3171	6	15.9	Pro->Hyp
219	QPGVPGP	95	650.3387	7	16.67	
220	GNPGPDGKLGPS	95	1094.5356	12	14.03	
221	VGPVF	98	517.29	5	35.8	
222	FXGVQ	98	562.2702	5	20.93	Pro->Hyp
223	VPGLP	97	481.29	5	22.83	
224	GPXGGYSGVP	97	902.4084	10	23.43	Pro->Hyp
225	LGLGPXG	95	625.3386	7	21.6	Pro->Hyp
226	XGGAR	98	472.2344	5	8.21	Pro->Hyp
227	FGLGXGAP	95	730.36	8	31.11	Pro->Hyp

228	APGPVGAP	98	664.3544	8	15.98	
229	LXGPQ	96	526.2701	5	11.02	Pro->Hyp
230	LGXGAL	95	542.3015	6	25.6	Pro->Hyp
231	VGPXGAP	98	609.3073	7	11.65	Pro->Hyp
232	VGPXGPA	96	609.3073	7	12.16	Pro->Hyp
233	SXGPDGKAGAP	99	968.4514	11	7.84	Pro->Hyp
234	FVGXGAP	95	659.3229	7	28.23	Pro->Hyp
235	GGFDLGAK	96	763.3864	8	23.17	
236	K(+42.01)XGLR	96	627.3655	5	18.76	Acetylation (N-term); Pro->Hyp
237	LXGPLQ	97	639.3542	6	22.38	Pro->Hyp
238	NXGPDKGLGSP	97	1053.5042	11	11.75	Pro->Hyp
239	GPLLK	99	526.3478	5	11.56	
240	AXGGLVGPK	97	810.455	9	12.57	Pro->Hyp
241	NAXGQDGRXGPXGGP	98	1420.6184	15	10.14	Pro->Hyp
242	NPGPDGKLGPS	96	1037.5142	11	14.06	
243	GPLGGAP	95	567.3016	7	14.83	
244	AVGPXGAP	98	680.3444	8	12.93	Pro->Hyp
245	APGVPGAP	98	664.3544	8	15.59	
246	AXGVPGAP	97	680.3444	8	13.47	Pro->Hyp
247	K(+42.01)AGPL	95	526.3115	5	29.39	Acetylation (N-term)
248	NXGPDGKLGSP	99	1053.5042	11	12.26	Pro->Hyp
249	LVGPXGP	99	651.3542	7	18.35	Pro->Hyp
250	FXGGKTGAYNPXGAP	95	1461.679	15	11.26	Pro->Hyp
251	LXGLA	95	485.28	5	23.77	Pro->Hyp
252	K(+42.01)VGPXGP	97	708.3757	7	23.71	Acetylation (N-term); Pro->Hyp
253	QVGPVF	95	645.3486	6	39.05	
254	A(+42.01)AVGPXGAP	95	793.392	9	22.56	Acetylation (N-term); Pro->Hyp
255	AGLGXGAP	96	654.3287	8	16	Pro->Hyp
256	QXGGQDGRSGPXGSP	97	1424.6182	15	8.32	Pro->Hyp
257	RVGPXGP	98	694.3713	7	10.55	Pro->Hyp
258	LXGGVP	97	554.3015	6	17.99	Pro->Hyp
259	LGLR	96	457.3012	4	12.56	
260	LAVGPXGAP	96	793.4285	9	22.27	Pro->Hyp
261	FGXGVQ	96	619.2916	6	23.83	Pro->Hyp
262	GPLGGVP	96	595.3329	7	23.22	
263	GPDGYSGPV	97	847.3712	9	23.75	
264	K(+42.01)GPVM	95	572.2992	5	24.93	Acetylation (N-term)
265	LLGPGXGAP	99	793.4285	9	23.32	Pro->Hyp
266	LXGLQ	97	542.3015	5	20.22	Pro->Hyp
267	NGFDLGAK	96	820.4079	8	22.75	
268	AXGGYSGVP	96	819.3713	9	21.66	Pro->Hyp
269	LVGPXGAP	98	722.3914	8	18.36	Pro->Hyp
270	LXGGLQ	95	599.3229	6	18.38	Pro->Hyp
271	LAGLR	95	528.3384	5	13.24	

272	LLGXGPGSP	96	809.4233	9	20.3	Pro->Hyp
273	FSGLDGAK	98	793.397	8	17.24	
274	LGXLAGPT	95	740.4019	8	27.13	Pro->Hyp
275	K(+42.01)GPVGP	96	595.3329	6	19.79	Acetylation (N-term)
276	LVGFQ	98	562.3115	5	29.12	
277	K(+42.01)GPVL	95	554.3428	5	31.73	Acetylation (N-term)
278	VGPVGP	95	524.2958	6	17.7	
279	LVGGLR	95	613.3911	6	13.73	
280	TENN(+.98)KN(+.98)FWLVK	98	1393.6877	11	38.95	Deamidation (NQ)
281	YSGPV	97	521.2485	5	16.65	
282	GLVGPK	96	569.3537	6	11.05	
283	RXGERGPXGPQ	95	1178.5693	11	7.34	Pro->Hyp
284	LALR	99	471.3169	4	11.68	
285	ALVGPK	97	583.3693	6	10.84	
286	NYSGVP	96	635.2914	6	19.8	
287	LVGPK	99	512.3322	5	8.47	
288	LLVGPK	96	625.4163	6	17.96	
289	VGPVGSP	98	611.3278	7	14.87	
290	AVLVGPK	95	682.4377	7	14.69	
291	QVGPVGSP	95	739.3864	8	17.94	
292	LTLR	96	501.3275	4	13	

X: Hyp

Table S2 The analysis certificate of gelatin supplied by Shanghai Xinxi Biotechnology Co.

**Test**	**Specification**	**Test result**	**Test Method**
**Physicochemical parameter**			
Transparency (10%solution)	≤25 NTU	≤21.31 NTU	Ref.TCVN 6184:2008
Appearance	Yellow to amber color powder	Yellow powder	Visual
Foreign material	Not detected	Not detected	Mental detector
pH value of 1% solution	5.0-7.0	5.42	USP
Gel strength (Bloom) 6.67%	250±15	243.1 g	USP
Viscosity (6.67%, 60℃)	≥ 30 mps (≥ 3.0 mPa.s)	35.6 mps (3.56 mPa.s)	GMIA
Moisture content	≤12%	9.31%	USP
(Residue on ignition) Total ash content	≤2%	0.27%	AOAC
Protein content	≥ 85%	89.2%	FAO
Particle size	≥ 95% through 8 mesh size	95.37% through 8 mesh	
Sulfit*	<3mg/kg	Pass	AOAC
Melamine	<2.5 ppm	Not detected	QQQ-LC/MS/MS-Ref FDA (USA)
**Heavy metal**			
Pb*	≤ 1.0 ppm	Pass	AOAC
As*	≤ 1.0 ppm	Pass	AOAC

Cr*	≤ 10 ppm	Pass	AOAC
**Microbiological parameter**			
Total Plate Count	≤ 1000 cfu/g	<10 cfu/g	USP
Total yeast and mould	<100 cfu/g	<10 cfu/g	USP
Escherichia Coli	Not detected	Not detected	USP
Coliforms	Not detected	Not detected	ISO 4831:2006
Salmonella	Negative/25g	Negative/25g	USP
**Hydroxyproline content**			
**7.258±0.089%**&			

*Performed on routine level, as per quality control plan.

& It was conducted using the alkali hydrolysis method with a commercially available kit (Jiangsu Kaiji Biotechnology Co. Nanjing, China)
